# Experiences and expert panel consensus on bridging therapy before anti-BCMA CAR-T cell therapy in multiple myeloma

**DOI:** 10.1038/s41409-026-02905-1

**Published:** 2026-05-13

**Authors:** Leo Rasche, Maria Theresa Krauth, Hermine Agis, Christoph Renner, Irene Strassl, Raphael Teipel, Vladan Vučinić, Katja Weisel, Wolfgang Willenbacher, Marc S. Raab

**Affiliations:** 1 University Hospital Würzburg, Würzburg, Germany; 2 Department of Internal Medicine I, Division of Hematology & Hemostaseology, Medical University of Vienna, Vienna, Austria; 3 Division of Hematology/Oncology Clinic, Hirslanden, Zurich, Switzerland; 4 Division of Hematology with Stem Cell Transplantation, Hemostaseology and Medical Oncology, Department of Internal Medicine I, Ordensklinikum Linz, Linz, Austria; 5 Medical Faculty, Johannes Kepler University Linz, Linz, Austria; 6 Department of Internal Medicine I, University Hospital Dresden, Technical University Dresden, Dresden, Germany; 7 Department of Hematology, Cellular Therapy, Hemostaseology and Infectious Diseases, University Hospital Leipzig, Leipzig, Germany; 8 Department of Hematology, Oncology and Bone Marrow Transplantation with Section of Pneumology, University Medical Center Hamburg-Eppendorf, Hamburg, Germany; 9 Internal Medicine V: Hematology and Oncology, Medical University of Innsbruck, Innsbruck, Austria; 10 Syndena GmbH, Connect to Cure, Innsbruck, Austria; 11 Heidelberg Myeloma Center, Department of Internal Medicine V, Heidelberg University Hospital and Medical Faculty, Heidelberg, Germany

**Keywords:** Cancer therapy, Cancer

## Abstract

Use of anti-B-cell maturation antigen (BCMA) chimeric antigen receptor T-cell (CAR-T) therapy to treat relapsed/refractory multiple myeloma is increasing. Studies suggest that effective bridging therapy (BT) prior to anti-BCMA CAR-T therapy can enhance efficacy and safety outcomes. Through qualitative interviews and a consensus workshop, 10 European experts shared their clinical experience regarding optimal BT selection, efficacy, safety and outcomes post–CAR-T, focussing on heavily pretreated patients and emerging BT options, such as bispecific T-cell engagers. Experts agreed that BT should aim to reduce tumour burden and maintain or improve patient performance status, while avoiding treatment-related toxicity that could delay or prevent CAR-T infusion. The balance between treatment duration and achieving an adequate response is important, and patient characteristics are key for BT selection, especially in difficult-to-treat populations. Here, we discuss the unique therapy talquetamab, a GPRC5DxCD3 bispecific antibody, which demonstrates robust efficacy and rapid response rates in clinical trials, and is being considered as a BT option before anti-BCMA CAR-T therapy based on expert experience and real-world data. This consensus, based on clinical experience, aims to provide guidance on BT for healthcare professionals (HCPs) involved in anti-BCMA CAR-T therapy and aid standardisation of care in this rapidly advancing field.

## Introduction

Chimeric antigen receptor T-cell (CAR-T) therapy has advanced the treatment landscape for patients with relapsed/refractory multiple myeloma (RRMM), providing high response rates and durable responses [1–3]. Two CAR-T therapies are currently approved in RRMM: ciltacabtagene autoleucel (cilta-cel) and idecabtagene vicleucel (ide-cel) [4, 5]. Cilta-cel is approved by the European Medicines Agency (EMA) and the US Food and Drug Administration (FDA) in patients who have received at least one prior line of therapy (LOT), including an immunomodulatory agent and a proteasome inhibitor (PI), and are lenalidomide-refractory, based on the CARTITUDE-4 trial [4, 6, 7]. Ide-cel is approved by the EMA and FDA for patients who have received two or more prior LOTs, including an immunomodulatory agent, a PI and an anti-CD38 monoclonal antibody (mAb), based on the KarMMa-3 trial [5, 8, 9]. In CARTITUDE-4, at a median follow-up of 33.6 months, median progression-free survival (PFS) was not reached (NR) in the cilta-cel group (hazard ratio [HR] versus standard of care [SOC], 0.29; 95% CI, 0.22–0.39); median overall survival (OS) was also NR with cilta-cel (HR versus SOC, 0.55; 95% CI, 0.39–0.79) [10]. In KarMMa-3, at a median follow-up of 30.9 months, median PFS was 13.8 months with ide-cel versus 4.4 months with SOC (HR, 0.49; 95% CI, 0.38–0.63); median OS was 41.4 months with ide-cel versus 37.9 months with SOC (HR, 1.01; 95% CI, 0.73–1.40) [11].

B-cell maturation antigen (BCMA)-targeting CAR-T therapies are manufactured from autologous T cells, genetically engineered to recognise BCMA on plasma cells [12]. Manufacturing is complex and typically takes 4–6 weeks [12–14], during which time disease control must be maintained to prevent progression and preserve patient fitness [13]. Bridging therapy (BT) is recommended to be administered between T-cell leukapheresis and lymphodepleting chemotherapy [13, 15]; however its use varies widely in practice [16]. BT options in earlier LOTs include chemotherapy, immunomodulatory drugs, PI, mAbs, and targeted agents [7, 9]. However, effective BTs in later LOTs remain limited [1, 2], prompting the exploration of novel therapies such as bispecific T-cell engagers in RRMM [17, 18].

In CARTITUDE-4, all patients in the cilta-cel group received BT with daratumumab, pomalidomide, and dexamethasone (DPd) or pomalidomide, bortezomib, and dexamethasone (PVd), with better BT responses correlating with longer PFS [19, 20]. KarMMa-3 allowed various BT combinations including PIs, immunomodulatory drugs and mAbs [9]. Patients who received BT and had a decrease/no change in disease burden had longer PFS than patients who had an increase in disease burden [20]. In early-phase trials (CARTITUDE-1 and KarMMa), low BT response rates were observed [1, 2], possibly due to heavy pretreatment (median 6 prior LOT), and use of novel therapies such as bispecific T-cell engagers was not permitted.

Real-world data show that effective BT before cilta-cel or ide-cel can minimise adverse events and improve outcomes in RRMM [18, 21]. While one study reports worse outcomes with ide-cel in patients who received alkylator-based BT versus those who did not, use of BT was more likely in patients with aggressive disease [22]. Additionally, intensity of cytotoxic chemotherapy BT has been associated with impaired hematopoietic recovery [23]. These data highlight the need for expert guidance to optimise BT selection, efficacy, safety, and post–CAR-T outcomes.

### Objective

This publication summarises the clinical experience and consensus recommendations of a panel of experts from multiple European centres proficient in managing RRMM with CAR-T therapy. We recognised a high need for guidance around optimal BT to CAR-T therapy in the European multiple myeloma (MM) community. Hence, we aim to provide guidance for BT before anti-BCMA CAR-T therapy, with particular focus on heavily pretreated patients and emerging BT options, such as bispecific T-cell engagers, to address current challenges and support harmonised clinical decision-making.

## Methods

A panel of 10 experts was assembled, with strong clinical backgrounds in haematology and experience in novel MM therapies, including CAR-T therapy. Within this, a steering committee guided the process to ensure consensus workshop objectives were met. Our approach involved qualitative individual interviews and a consensus workshop, both focused on BT in RRMM, including bispecific T-cell engagers as BT options before anti-BCMA CAR-T therapy.

### Qualitative interviews

In January 2025, three steering committee members developed interview questions addressing BT concepts and options for later treatment lines. Topics selected were areas where practical guidance would be highly beneficial to healthcare professionals (HCPs) using BT in anti-BCMA CAR-T therapy. One-hour individual interviews were conducted to capture uninfluenced opinions. Results were summarised in April 2025 to prepare for the workshop.

### Consensus workshop

In the workshop, we reviewed interview insights and resolved areas lacking consensus, covering topics like BT definition and objectives, patient eligibility, balancing duration and response, regimen selection, difficult-to-treat groups, and clinical and safety management. We discussed evolving practices, including shifting from minimising BT duration to optimising the balance between duration and response to BT.

The virtual meeting used structured dialogue and voting to encourage active participation and discussion, which was consolidated into key expert recommendations. While our guidance offers flexible direction based on our experience and emerging evidence, it does not replace clinical judgment or legal advice.

## Expert guidance and opinions: BT in anti-BCMA CAR-T therapy

### Scope of the recommendations

For the purposes of this publication, BT was defined as therapy administered after leukapheresis and before lymphodepleting chemotherapy preceding anti-BCMA CAR-T infusion, in alignment with previous publications [13, 24, 25]. It was deemed important to distinguish between BT and pre-bridging/holding treatments, defined as treatments administered before apheresis [13, 18]. Treatment goals and considerations may be different for BT and pre-bridging/holding treatments. This publication focuses on BT before anti-BCMA CAR-T therapy in triple-class exposed patients with RRMM.

### Recipients of BT

The recent International Myeloma Working Group (IMWG) guidelines recommend consideration of BT in patients with high disease burden and/or at risk of developing morbidity from MM during T-cell manufacturing [13]. Based on our experience, BT should be used for a vast majority of patients undergoing anti-BCMA CAR-T therapy, regardless of prior LOTs; effective BT enables most patients to receive CAR-T infusion. Patients experiencing smouldering relapse (typically in earlier LOTs) or slowly progressing disease will still benefit from BT. In exceptional circumstances, for example where contraindications or substantial comorbidities are present, BT may not be administered; however, such decisions would be made on an individual basis rather than reflecting a clearly defined population unsuitable for BT.

Although anticipated future reductions in vein-to-vein time may lessen the need for BT in some patients, it should still be considered an opportunity to reduce tumour load before infusion, particularly as effective BT has been associated with improved efficacy and safety outcomes of CAR-T therapy [26, 27].

### Objectives of BT

We agreed that the short-term objectives of BT are to: Bring patients to anti-BCMA CAR-T therapy, maintain or improve performance status and organ function until CAR-T infusion and reduce tumour burden, while avoiding treatment-related toxicity and worsening cytopenia that could delay or prevent CAR-T infusion. Long-term objectives include maximising efficacy post–CAR-T therapy and reducing the risk of post–CAR-T toxicities, as high tumour burden is a major risk factor for development of typical CAR-T associated toxicities (cytokine-release syndrome [CRS], immune effector cell-associated neurotoxicity syndrome [ICANS] and non-ICANS neurotoxicities) [28]. These objectives are supported by literature [13, 24, 29–32]. Available evidence suggests effective BT is associated with improved efficacy and safety of subsequent CAR-T therapy [18–22, 27]. Within these objectives, the optimal balance between treatment duration and achieving an adequate response must be considered.

### Balancing duration and response

Although data on optimal duration of BT in CAR-T therapy are limited [25], HCPs should balance duration with achieving an adequate response. BT should last as ‘short as possible’ and ‘as long as necessary’ to reach the deepest response without delaying or preventing CAR-T infusion. We generally agreed that reaching ≥complete response during BT is not always possible; ≥partial response is an appropriate target. In patients with low tumour burden, however, this may not be required – a lower level of response and absence of progressive disease might be sufficient.

In some cases, extending BT to optimise response may be considered if a deeper response is achievable, though no consensus exists on extending BT by 1–2 or >2 cycles. Substantially prolonged BT is discouraged due to risks of disease progression and infections, which may delay or prevent CAR-T infusion. Patients should proceed to CAR-T infusion if CAR-T manufacturing was successful, even if achieving a deep response (e.g., ≥very good partial response) during BT. Extension of BT to optimise response should follow careful monitoring of disease and toxicity parameters. Guidance priorities are:

- 1: Stop proliferative disease and gain some response.
- 2: Add 1 cycle if good response occurs rapidly.

We agreed that patients who do not achieve ≥partial response after initial BT should switch to a different (second-line) BT regimen; the objectives of this may differ. In this context, attaining minor response or control of stable disease may be a reasonable goal, aiming to preserve eligibility for CAR-T and avoid further progression of disease. Importantly, patients with rapid disease progression or high/uncontrolled tumour burden on BT may not be recommended for CAR-T.

### Choice of BT

The literature shows heterogeneity in BT options [23, 24], reflecting differences in prior LOTs, patient factors (e.g., age and cytogenetics) and status (e.g., extramedullary disease [EMD], renal impairment, central nervous system [CNS] involvement, bone marrow reserve, and cytopenias/infections risk), tumour dynamics, and drug availability. Patient characteristics and prior therapies should guide BT selection. Patients with progressive disease on BT should be promptly evaluated for alternative BT regimens, depending on availability/access and prior treatments, to avoid delay or prevention of CAR-T infusion. Typically, more BT options are available in earlier lines; effective BT is more challenging in heavily pretreated patients. BTs that have been most frequently used in RRMM after ≥3 prior LOTs include chemotherapy, immunomodulatory drugs, PI, monoclonal antibodies, targeted inhibitors (BCL-2 and XPO1), and talquetamab (Table 1).

**Table 1:** Bridging therapy options in CAR-T therapy in patients with RRMM who have received ≥3 lines of therapy^a^.

Chemotherapy combinations	mAb combinations	PI combinations	Targeted inhibitors (BCL-2 and XPO1) and combinations	Unique/Novel therapies: Bispecific antibodies
Cisplatin + doxorubicin + cyclophosphamide + etoposide (PACE)-like therapy [58]	Anti-CD38 mAb + pomalidomide + dexamethasone	Carfilzomib + dexamethasone	Venetoclax [1, 13]	Talquetamab [18, 37]
Dexamethasone + cisplatin + doxorubicin + cyclophosphamide + etoposide (DPACE) [58]	Anti-CD38 mAb + carfilzomib + dexamethasone	Carfilzomib + Anti-CD38 mAb + dexamethasone	Selinexor [13]	
Dexamethasone + cyclophosphamide + etoposide + cisplatin (DCEP) [53]	Anti-CD38 mAb + PI + immunomodulatory drug + dexamethasone	Carfilzomib + cyclophosphamide + dexamethasone [53]	Selinexor + bortezomib or pomalidomide	
Cyclophosphamide in combination [1, 2]	Elotuzumab + pomalidomide + dexamethasone	Carfilzomib + cyclophosphamide + Anti-CD38 mAb	BRAF inhibitors +/- MEK inhibitor	
Cyclophosphamide + etoposide + dexamethasone		Carfilzomib + pomalidomide + Anti-CD38 mAb		
Bendamustine [1]		Carfilzomib + pomalidomide + isatuximab		
Melphalan [1]		Bortezomib + dexamethasone [53]		

*BCL-2* B-cell lymphoma 2, *CAR-T* chimeric antigen receptor T-cell, *mAb* monoclonal antibodies, *PI* proteasome inhibitor, *RRMM* relapsed/refractory multiple myeloma, *XPO1* exportin 1.

^a^The bridging therapy categories are listed alphabetically.

In patients who do not achieve ≥partial response to initial BT, treatment decisions should be guided by treatment history and patient characteristics.

Additional considerations for regimen selection include:

- Avoid agents to which the patient was previously refractory.
- Prioritise conventional RRMM therapies; consider prior exposure and refractory status in triple-class exposed patients.
- Consider mAb-based regimens in patients with limited exposure/prolonged period since exposure.
- Consider high-dose melphalan and DPACE (dexamethasone + cisplatin + doxorubicin + cyclophosphamide + etoposide) only for fit, non-elderly patients who have exhausted most options and have sufficient bone marrow reserve and/or an autologous back-up available.
- Consider chemotherapy-based regimens for patients with high tumour burden and/or EMD.
- Consider talquetamab in heavily pretreated patients who have exhausted other effective therapies.
- Avoid anti-BCMA BT before anti-BCMA CAR-T therapy due to unknown effects of short-term use and resistance concerns.
- Consider local reimbursement policies and accessibility; no regimen is FDA or EMA-approved specifically for BT and available treatments differ widely by country.

### Choice of BT – Talquetamab

Talquetamab, a GPRC5DxCD3 bispecific antibody, is EMA-approved for patients with RRMM who have received at least three prior therapies, including an immunomodulatory agent, a PI, and an anti-CD38 mAb, and have demonstrated disease progression on the last therapy [33], and FDA-approved after at least four prior lines of therapy, including a PI, an immunomodulatory agent, and an anti-CD38 mAb [34]. In MonumenTAL-1, talquetamab demonstrated a high ORR of 67–74%, median duration of response of 17.5 months at a dosing schedule of 0.8 mg/kg every other week [35], and rapid responses with a median time to first response of 1.2 and 1.3 for 0.4 mg/kg every week and 0.8 mg/kg every other week, respectively [36]. The safety profile showed lower risk of high-grade infections versus approved anti-BCMA bispecific antibodies [35].

Based on the favourable efficacy outcomes and rapid response rates in MonumenTAL-1 [35, 36], we recommend considering talquetamab as BT, particularly for patients who have exhausted other effective treatments. Retrospective studies from multiple centres in Germany and the US also support the efficacy and manageable safety of talquetamab as BT before anti-BCMA CAR-T therapy [17, 18, 37]. These studies reported ORRs of talquetamab BT at 62–100% [17, 18, 37], with talquetamab outperforming chemotherapy or other antibody-based regimens [17], enabling most patients to successfully proceed to CAR-T infusion [17, 18, 37].

Patients eligible for CAR-T therapy may be considered eligible for bridging with talquetamab, considering their characteristics, pre-existing conditions, and clinically relevant impairments such as taste changes, skin conditions, or weight loss. As supported by recent findings [18], talquetamab is favourable for difficult-to-treat groups including renal impairment, advanced age, high disease burden, functional high-risk status, or EMD, especially since patients with EMD often do not respond well to other therapies.

Consistent with other BT regimens, the typical duration of talquetamab as BT is 6–8 weeks. We noted safety considerations with talquetamab including those related to GPRC5D targeting, such as early-onset taste changes and skin reactions, which rarely lead to discontinuation but require supportive care [36–39]. Nail toxicity typically occurs later (median onset of 64–69 days) [38] and is less relevant during bridging. CRS and ICANS are expected, with ICANS seen in <10% of cases and mostly low grade; delayed neurotoxicity is rare (<2%) [17, 18, 37, 38].

Bispecific antibodies like talquetamab may serve as salvage options after anti-BCMA CAR-T relapse [40], but data on reuse in patients who previously received it as BT is lacking. We would consider reusing talquetamab after CAR-T progression if a non-BCMA targeting agent is needed as alternatives are limited, especially if there was a prior response to talquetamab. Efficacy may be reduced shortly after CAR-T therapy, highlighting the need for prospective studies to guide clinical practice.

### BT in difficult-to-treat patient groups

#### Patients with EMD

In the pivotal cilta-cel and ide-cel trials, EMD incidence in patients with RRMM was 13–39% [2, 7, 9, 41]. In general, studies have shown negative correlations between EMD and treatment outcomes in patients with RRMM [16, 42–44]. Although superior outcomes were observed across all patient subgroups (including EMD) with CAR-T therapy vs SOC [9, 10], studies such as LocoMMotion showed worse PFS outcomes in patients with versus without EMD (median 2.7 vs 5.1 months) [42]. In addition to systemic treatment, we recommend considering radiation as BT in RRMM with EMD (depending on tumour location and size), painful osteolytic lesions, or risk of fracture. One study found this approach safe and feasible in patients with EMD [45]. Talquetamab may also be considered for BT in these patients [18]. EMD should be controlled when CAR-T therapy is administered.

#### Patients eligible for molecular-driven therapies

The t(11;14) chromosomal translocation, present in 16–24% MM cases, represents the most common primary translocation in plasma cell disorders [46]. There are no approved mutation-targeted agents for MM; however, venetoclax, a BCL-2 inhibitor, is suggested in recent IMWG recommendations for patients with RRMM with the t(11;14) translocation, though its off-label use should be guided by local regulatory frameworks and clinical judgment [13]. This is particularly relevant to patients with limited treatment options.

BRAF V600E is the most common BRAF mutation, seen in ~4% of newly diagnosed MM and ~8% of RRMM [47–50]. BRAF inhibitors, potentially in combination with a MEK inhibitor, may be a BT option for patients harbouring BRAF mutations, who have limited treatment options.

#### Patients with CNS involvement

CNS involvement, a rare form of EMD, affects <1% of patients with MM [51, 52]. Although there are concerns that anti-BCMA CAR-T therapy could increase the incidence or severity of neurotoxicity including ICANS, a recent study suggests that CAR-T therapy in MM with CNS involvement is safe and feasible [53]. Here, patients were given BT similar to our recommendations [53]. We recommend considering various options including intrathecal therapy (e.g., dexamethasone, methotrexate and/or cytarabine), combination of selinexor and pomalidomide (repeated until clearance of plasma cells in the CNS), chemotherapy combination (methotrexate, dexamethasone, and cytarabine-arabinoside), with or without radiation therapy, or PACE-based regimens (cisplatin + doxorubicin + cyclophosphamide + etoposide) [54, 55].

### Clinical management

In our experience, duration of BT ranges from 6–8 weeks (i.e., 1–2 cycles), which is generally sufficient to induce meaningful disease control. If feasible, we recommend an approximate 2-week wash-out period between BT and lymphodepleting chemotherapy, regardless of BT regimen.

In clinical practice, obtaining a deep response (≥very good partial response) during the typical BT (1–2 cycles) period is not always possible; similarly, full response assessment work-up according to IMWG guidelines [15] may not be feasible. Thus, from a practical perspective, achieving ≥partial response may be a reasonable treatment goal with BT; such responses may be assessed by measuring serum and urine M-protein, in combination with imaging (to assess EMD before CAR-T infusion). This is supported by a recent analysis of the CARTITUDE-4 study showing that ≥25% paraprotein reduction from baseline after BT was associated with longer PFS post–cilta-cel versus <25% [19] and findings from a real-world cohort from Germany, where BT success correlated with safety and PFS after CAR-T therapy, regardless of whether patients received ide-cel or cilta-cel [21].

### Safety consideration

Ongoing infections or organ toxicities may delay or prohibit CAR-T infusion in patients with RRMM [7, 9]. Minimising toxicity risk during BT is paramount to avoiding any delay or prevention of CAR-T infusion. This includes avoiding excessive haematotoxicity, which may delay lymphodepletion or increase infection risk, awareness of effective management of specific adverse events associated with bridging agents, and monitoring and preventing infections. The CAR-HEMATOTOX score, a tool used to predict haematological toxicity in patients receiving CAR-T therapy [56], may help with risk assessment and BT regimen selection. However, a high CAR-HEMATOTOX score should not prevent CAR-T therapy. Based on our clinical observations, haematotoxic BT should be avoided in patients with high CAR-HEMATOTOX scores.

## Conclusions

BT is a vital component of anti-BCMA CAR-T therapy in RRMM, requiring careful balance of efficacy, safety, and timing. HCPs should aim for the deepest possible response without risking any delay or prevention of CAR-T infusion. This consensus provides guidance on selecting BTs tailored to patients and their disease characteristics, and highlights potential challenges, particularly in heavily pre-treated patients. Analyses of talquetamab as BT in real-world clinical practice have generated promising findings, aligned with our clinical experience. Additionally, recently published EMN guidance considers talquetamab a valid BT option for patients who have exhausted other treatments [57]. Our consensus is based on clinical experience and is intended to serve as a reference for HCPs using anti-BCMA CAR-T therapy in RRMM. Continued research and shared clinical experience will enhance standardisation and optimise care delivery in this rapidly evolving field.

## Data Availability

The data sharing policy of Johnson & Johnson Innovative Medicine is available at https://www.janssen.com/clinical-trials/transparency. Requests for access to the study data can be submitted through Yale Open Data Access (YODA) Project site at http://yoda.yale.edu.

## References

[1] Berdeja JG, Madduri D, Usmani SZ, Jakubowiak A, Agha M, Cohen AD, et al. Ciltacabtagene autoleucel, a B-cell maturation antigen-directed chimeric antigen receptor T-cell therapy in patients with relapsed or refractory multiple myeloma (CARTITUDE-1): a phase 1b/2 open-label study. Lancet. 2021;398:314–24. 34175021 10.1016/S0140-6736(21)00933-8

[2] Munshi NC, Anderson Jr LD, Shah N, Madduri D, Berdeja J, Lonial S, et al. Idecabtagene vicleucel in relapsed and refractory multiple myeloma. N Engl J Med. 2021;384:705–16. 33626253 10.1056/NEJMoa2024850

[3] Lin Y, Martin TG, Usmani SZ, Berdeja JG, Jakubowiak AJ, Agha ME, et al. CARTITUDE-1 final results: Phase 1b/2 study of ciltacabtagene autoleucel in heavily pretreated patients with relapsed/refractory multiple myeloma. Am Soc Clin Oncol. 2023;7:e6102468.

[4] EMA. Carvykti (ciltacabtagene autoleucel). Summary of product characteristics. Belgium: Janssen-Cilag International NV; 2024.

[5] EMA. Abecma (idecabtagene vicleucel). Summary of product characteristics, 2022 ed. Dublin: Bristol Myers Squibb; 2025.

[6] FDA. CARVYKTI^®^ (ciltacabtagene autoleucel) prescribing information. Horsham, PA 19044, USA: Janssen Biotech, Inc; 2025.

[7] San-Miguel J, Dhakal B, Yong K, Spencer A, Anguille S, Mateos M-V, et al. Cilta-cel or standard care in lenalidomide-refractory multiple myeloma. N Engl J Med. 2023;389:335–47. 37272512 10.1056/NEJMoa2303379

[8] FDA. ABECMA^®^ (idecabtagene vicleucel) prescribing information. In: Summit, NJ 07901: Celgene Corporation, a Bristol-Myers Squibb Company; 2025.

[9] Rodriguez-Otero P, Ailawadhi S, Arnulf B, Patel K, Cavo M, Nooka AK, et al. Ide-cel or standard regimens in relapsed and refractory multiple myeloma. N Engl J Med. 2023;388:1002–14. 36762851 10.1056/NEJMoa2213614

[10] Overall survival with ciltacabtagene autoleucel versus standard of care in lenalidomide-refractory multiple myeloma: phase 3 CARTITUDE-4 study update. In: 21st International Myeloma Society Annual Meeting. Rio de Janeiro, Brazil; 2024.

[11] Idecabtagene vicleucel versus standard regimens in patients with triple-class-exposed relapsed and refractory multiple myeloma: updated analysis from KarMMa-3. In 65th ASH Annual Meeting and Exposition. San Diego, California; 2023.

[12] Ailawadhi S, Shune L, Wong SW, Lin Y, Patel K, Jagannath S, et al. Optimizing the CAR T-cell therapy experience in multiple myeloma: Clinical pearls from an expert roundtable. Clin Lymphoma Myeloma. 2024;24:e217–e225. 10.1016/j.clml.2024.01.014 38369437

[13] Costa LJ, Banerjee R, Mian H, Weisel K, Bal S, Derman BA, et al. International myeloma working group immunotherapy committee recommendation on sequencing immunotherapy for treatment of multiple myeloma. Leukemia. 2025;39:543–54. 39870767 10.1038/s41375-024-02482-6 PMC11879857

[14] Al Hadidi S, Szabo A, Esselmann J, Hammons L, Hussain M, Ogunsesan Y, et al. Clinical outcome of patients with relapsed refractory multiple myeloma listed for BCMA directed commercial CAR-T therapy. Bone Marrow Transpl. 2023;58:443–5. 10.1038/s41409-022-01905-1 36550200

[15] Lin Y, Qiu L, Usmani S, Joo CW, Costa L, Derman B, et al. Consensus guidelines and recommendations for the management and response assessment of chimeric antigen receptor T-cell therapy in clinical practice for relapsed and refractory multiple myeloma: a report from the International Myeloma Working Group Immunotherapy Committee. Lancet Oncol. 2024;25:e374–e387. 38821074 10.1016/S1470-2045(24)00094-9

[16] Sidana S, Patel KK, Peres LC, Bansal R, Kocoglu MH, Shune L, et al. Safety and efficacy of standard-of-care ciltacabtagene autoleucel for relapsed/refractory multiple myeloma. Blood. 2025;145:85–97. 39365257 10.1182/blood.2024025945 PMC11952008

[17] Fandrei D, Seiffert S, Rade M, Rieprecht S, Gagelmann N, Born P, et al. Bispecific antibodies as bridging to BCMA CAR-T cell therapy for relapsed/refractory multiple myeloma. Blood Cancer Discov. 2025;6:38–54. 39441177 10.1158/2643-3230.BCD-24-0118 PMC11707513

[18] Dhakal B, Akhtar OS, Fandrei D, Jensen A, Banerjee R, Pan D, et al. Sequential targeting in multiple myeloma: talquetamab, a GPRC5D bispecific antibody, as a bridge to BCMA CAR-T therapy. Blood. 2025;146:2063–72. 40749169 10.1182/blood.2025029773

[19] Effectiveness of bridging therapy corresponds to improved outcomes after receiving CAR-T therapy: phase 3 CARTITUDE-4 study of patients with relapsed, lenalidomide-refractory multiple myeloma. In: 21st International Myeloma Society (IMS) Annual Meeting. Rio de Janeiro, Brazil; 2024.

[20] Einsele H, Rodriguez-Otero P, Arnulf B, Patel K, Manier S, Costa L, et al. P-008 Idecabtagene vicleucel (ide-cel) vs standard regimens in triple-class–exposed (TCE) relapsed and refractory multiple myeloma (RRMM): KarMMa-3 subgroup analysis in patients receiving bridging therapy. Clin Lymphoma Myeloma Leuk. 2023;23:S37 10.1016/S2152-2650(23)01626-9.

[21] Merz M, Gagelmann N, Smaili S, Flossdorf S, Sauer S, Scheid C et al. Remission conversion drives outcomes after CAR T-cell therapy for multiple myeloma: a registry analysis from the DRST. Blood. 2025;146:1677–86. 10.1182/blood.2025028330 40504993

[22] Afrough A, Hashmi H, Hansen DK, Sidana S, Ahn C, Peres LC, et al. Real-world impact of bridging therapy on outcomes of ide-cel for myeloma in the US Myeloma Immunotherapy Consortium. Blood Cancer J. 2024;14:63. 38609386 10.1038/s41408-024-00993-0 PMC11015040

[23] Frenking JH, Zhou X, Rejeski K, Wagner V, Costello P, Hielscher T, et al. Bridging intensity is associated with impaired hematopoietic recovery after BCMA CAR-T therapy for multiple myeloma. Blood Adv. 2025;9:4151–66. 10.1182/bloodadvances.2024015732. 40267180 10.1182/bloodadvances.2024015732 PMC12361772

[24] Bhaskar ST, Dholaria B, Savani BN, Oluwole O. The evolving role of bridging therapy during CAR-T therapy. Clin Hematol Int. 2024;6:9. 39027142 10.46989/001c.116261 PMC11257384

[25] Kaestner V, Haslam A, Prasad V. Characterization of bridging therapies in clinical trials leading to FDA approval of CAR-T cell therapies. Int J Cancer. 2025;157:1376–85. 10.1002/ijc.35473 40394455

[26] Effectiveness of bridging therapy corresponds to improved outcomes after ciltacabtagene autoleucel: phase 3 CARTITUDE-4 study of patients with relapse, lenalidomide-refractory multiple myeloma. In: 67th ASH Annual Meeting and Exposition. Orlando, Florida; 2025.

[27] Bridging therapy response and low pre-lymphodepletion plasma cell burden are associated with improved safety and efficacy outcomes of ciltacabtagene autoleucel in multiple myeloma. In: 67th ASH Annual Meeting and Exposition. Orlando, Florida; 2025.

[28] Swan D, Madduri D, Hocking J. CAR-T cell therapy in Multiple Myeloma: current status and future challenges. Blood Cancer J. 2024;14:206. 10.1038/s41408-024-01191-8. 39592597 10.1038/s41408-024-01191-8 PMC11599389

[29] Cohen AD, Parekh S, Santomasso BD, Gállego Pérez-Larraya J, van de Donk NW, Arnulf B, et al. Incidence and management of CAR-T neurotoxicity in patients with multiple myeloma treated with ciltacabtagene autoleucel in CARTITUDE studies. Blood Cancer J. 2022;12:32. 35210399 10.1038/s41408-022-00629-1 PMC8873238

[30] Bal S, Costa LJ. Bridging treatment prior to chimeric antigen receptor T-cell therapy in multiple myeloma. Br J Haematol. 2024;204:449–54. 38036424 10.1111/bjh.19227

[31] Bhaskar ST, Dholaria BR, Sengsayadeth SM, Savani BN, Oluwole OO. Role of bridging therapy during chimeric antigen receptor T cell therapy. EJHaem. 2022;3:39–45. 35844303 10.1002/jha2.335 PMC9175845

[32] Richter J. Like a bridge over troubled water: keeping the myeloma down en route to CAR-T. Blood Cancer J. 2024;14:64. 38609377 10.1038/s41408-024-01049-z PMC11015014

[33] EMA Talvey (Talquetamab). Summary of product characteristics. Belgium: Janssen-Cilag International NV; 2023.

[34] FDA Talvey TM (Talquetamab-Tgvs) Prescribing Information. 2023.

[35] Rasche L, Schinke CD, Touzeau C, Minnema M, Van De Donk NW, Rodríguez-Otero P et al. Efficacy and safety from the phase 1/2 MonumenTAL-1 study of talquetamab, a GPRC5D× CD3 bispecific antibody, in patients with relapsed/refractory multiple myeloma: analyses at an extended median follow-up. Am Soc Clin Oncol. 2025;43:7528.

[36] P915: long-term efficacy and safety results from the phase 1/2 MonumenTAL-1 study of talquetamab, a GPRC5D× CD3 bispecific antibody, in patients with relapsed/refractory multiple myeloma (RRMM). Madrid, Spain: European Hematology Association (EHA); 2024.

[37] Dhakal B, Akhtar OS, Cowan AJ, Richard S, Friend R, Rees MJ, et al. Talquetamab bridging: paving the way to B-cell maturation antigen (BCMA) CAR-T cell therapy in relapsed/refractory multiple myeloma (RRMM). Blood. 2024;144:931.38905596

[38] Chari A, Krishnan A, Rasche L, Ye JC, Garfall A, Popat R, et al. Clinical management of patients with relapsed/refractory multiple myeloma treated with talquetamab. Clin Lymphoma Myeloma Leuk. 2024;24:665–93. 38871558 10.1016/j.clml.2024.05.003

[39] Liu L, Krishnan A. Talquetamab in multiple myeloma. Haematologica. 2023;109:718. 10.3324/haematol.2023.283931 PMC10905093 37855056

[40] Updated results of talquetamab, a GPRC5D× CD3 bispecific antibody, in patients with relapsed/refractory multiple myeloma with prior exposure to T-cell redirecting therapies: results of the phase 1/2 MonumenTAL-1 study. In: 65th American Society of Hematology (ASH) Annual Meeting. San Diego, California; 2023.

[41] Martin T, Usmani SZ, Berdeja JG, Agha M, Cohen AD, Hari P, et al. Ciltacabtagene autoleucel, an anti–B-cell maturation antigen chimeric antigen receptor T-cell therapy, for relapsed/refractory multiple myeloma: CARTITUDE-1 2-year follow-up. J Clin Oncol. 2023;41:1265–74. 35658469 10.1200/JCO.22.00842 PMC9937098

[42] Mateos M-V, Weisel K, De Stefano V, Goldschmidt H, Delforge M, Mohty M, et al. LocoMMotion: a study of real-life current standards of care in triple-class exposed patients with relapsed/refractory multiple myeloma–2-year follow-up (final analysis). Leukemia. 2024;38:2554–60. 39322709 10.1038/s41375-024-02404-6 PMC11588650

[43] Zanwar S, Sidana S, Shune L, Puglianini OC, Pasvolsky O, Gonzalez R, et al. Impact of extramedullary multiple myeloma on outcomes with idecabtagene vicleucel. J Hematol Oncol. 2024;17:42. 38845015 10.1186/s13045-024-01555-4 PMC11157748

[44] Dima D, Abdallah A-O, Davis JA, Awada H, Goel U, Rashid A, et al. Impact of extraosseous extramedullary disease on outcomes of patients with relapsed-refractory multiple myeloma receiving standard-of-care chimeric antigen receptor T-cell therapy. Blood Cancer J. 2024;14:90. 38821914 10.1038/s41408-024-01068-w PMC11143360

[45] Manjunath SH, Cohen AD, Lacey SF, Davis MM, Garfall AL, Melenhorst JJ, et al. The safety of bridging radiation with anti-BCMA CAR T-cell therapy for multiple myeloma. Clin Cancer Res. 2021;27:6580–90. 34526365 10.1158/1078-0432.CCR-21-0308 PMC8639780

[46] Bal S, Kumar SK, Fonseca R, Gay F, Hungria VT, Dogan A, et al. Multiple myeloma with t (11; 14): unique biology and evolving landscape. Am J Cancer Res. 2022;12:2950. 35968339 PMC9360221

[47] Xu J, Pfarr N, Endris V, Mai EK, Md Hanafiah N, Lehners N, et al. Molecular signaling in multiple myeloma: association of RAS/RAF mutations and MEK/ERK pathway activation. Oncogenesis. 2017;6:e337–e337. 28504689 10.1038/oncsis.2017.36 PMC5523069

[48] Andrulis M, Lehners N, Capper D, Penzel R, Heining C, Huellein J, et al. Targeting the BRAF V600E mutation in multiple myeloma. Cancer Discov. 2013;3:862–9. 23612012 10.1158/2159-8290.CD-13-0014

[49] John L, Krauth MT, Podar K, Raab M-S. Pathway-directed therapy in multiple myeloma. Cancers. 2021;13:1668. 33916289 10.3390/cancers13071668 PMC8036678

[50] Sharman J, Chmielecki J, Morosini D, Palmer G, Ross J, Stephens P, et al. Vemurafenib response in 2 patients with posttransplant refractory BRAF V600E–mutated multiple myeloma. Clin Lymphoma Myeloma Leuk. 2014;14:e161–e163. 24997557 10.1016/j.clml.2014.06.004

[51] Egan PA, Elder PT, Deighan WI, O’Connor SJ, Alexander HD. Multiple myeloma with central nervous system relapse. Haematologica. 2020;105:1780. 32414852 10.3324/haematol.2020.248518 PMC7327654

[52] Sammartano V, Cerase A, Venanzi V, Mazzei MA, Vangone BE, Gentili F, et al. Central nervous system myeloma and unusual extramedullary localizations: real life practical guidance. Front Oncol. 2022;12:934240. 35875104 10.3389/fonc.2022.934240 PMC9300839

[53] Gaballa MR, Puglianini OC, Cohen A, Vogl D, Chung A, Ferreri CJ, et al. BCMA-directed CAR T-cell therapy in patients with multiple myeloma and CNS involvement. Blood Adv. 2025;9:1171–80. 39729503 10.1182/bloodadvances.2024014345 PMC11925525

[54] Ghandili S, Alihodzic D, Wiessner C, Bokemeyer C, Weisel K, Leypoldt LB. VTd-PACE and VTd-PACE-like regimens are effective salvage therapies in difficult-to-treat relapsed/refractory multiple myeloma: a single-center experience. Ann Hematol. 2023;102:117–24. 10.1007/s00277-022-05027-y. 36383242 10.1007/s00277-022-05027-y PMC9667441

[55] Huynh T, Corre E, Lemonnier MP, Dulery R, Marjanovic Z, Jaff N, et al. Role of D(T)PACE-based regimens as treatment of multiple myeloma with extramedullary relapse or refractory disease. Leuk Lymphoma. 2021;62:2235–41. 10.1080/10428194.2021.1907373. 33792474 10.1080/10428194.2021.1907373

[56] Rejeski K, Hansen DK, Bansal R, Sesques P, Ailawadhi S, Logue JM, et al. The CAR-HEMATOTOX score as a prognostic model of toxicity and response in patients receiving BCMA-directed CAR-T for relapsed/refractory multiple myeloma. J Hematol Oncol. 2023;16:88. 37525244 10.1186/s13045-023-01465-x PMC10391746

[57] Van de Donk N, Moreau P, San Miguel JF, Mateos MV. Sequencing BCMA- and GPRC5D-targeting immunotherapies in multiple myeloma: practical guidance from the European Myeloma Network. HemaSphere. 2025;9. 10.1002/hem3.70260 10.1002/hem3.70260 PMC12645799 41306326

[58] Parrondo RD, Roy V, Sher T, Alegria V, Chanan-Khan AA, Ailawadhi S. Use of KRD-PACE as salvage therapy in aggressive, relapsed/bortezomib-refractory extramedullary multiple myeloma: a report of two cases and literature review. Case Rep Hematol. 2020;2020:4360926. 32148978 10.1155/2020/4360926 PMC7049436

